# Utility of a Mouse Model of Osteoarthritis to Demonstrate Cartilage Protection by IFNγ-Primed Equine Mesenchymal Stem Cells

**DOI:** 10.3389/fimmu.2016.00392

**Published:** 2016-09-27

**Authors:** Marie Maumus, Gautier Roussignol, Karine Toupet, Geraldine Penarier, Isabelle Bentz, Sandrine Teixeira, Didier Oustric, Mireille Jung, Olivier Lepage, Regis Steinberg, Christian Jorgensen, Danièle Noel

**Affiliations:** ^1^U1183, INSERM, Hôpital Saint-Eloi, Montpellier, France; ^2^Montpellier University, UFR de Médecine, Montpellier, France; ^3^Sanofi, Montpellier, France; ^4^GREMERES-ICE, University of Lyon, Marcy l’Etoile, France; ^5^Clinical Immunology and Osteoarticular Diseases Therapeutic Unit, Hôpital Lapeyronie, Montpellier, France

**Keywords:** mesenchymal stem cells, osteoarthritis, cell therapy, cartilage, secretome, horse

## Abstract

**Objective:**

Mesenchymal stem cells isolated from adipose tissue (ASC) have been shown to influence the course of osteoarthritis (OA) in different animal models and are promising in veterinary medicine for horses involved in competitive sport. The aim of this study was to characterize equine ASCs (eASCs) and investigate the role of interferon-gamma (IFNγ)-priming on their therapeutic effect in a murine model of OA, which could be relevant to equine OA.

**Methods:**

ASC were isolated from subcutaneous fat. Expression of specific markers was tested by cytometry and RT-qPCR. Differentiation potential was evaluated by histology and RT-qPCR. For functional assays, naïve or IFNγ-primed eASCs were cocultured with peripheral blood mononuclear cells or articular cartilage explants. Finally, the therapeutic effect of eASCs was tested in the model of collagenase-induced OA (CIOA) in mice.

**Results:**

The immunosuppressive function of eASCs on equine T cell proliferation and their chondroprotective effect on equine cartilage explants were demonstrated *in vitro*. Both cartilage degradation and T cell activation were reduced by naïve and IFNγ-primed eASCs, but IFNγ-priming enhanced these functions. In CIOA, intra-articular injection of eASCs prevented articular cartilage from degradation and IFNγ-primed eASCs were more potent than naïve cells. This effect was related to the modulation of eASC secretome by IFNγ-priming.

**Conclusion:**

IFNγ-priming of eASCs potentiated their antiproliferative and chondroprotective functions. We demonstrated that the immunocompetent mouse model of CIOA was relevant to test the therapeutic efficacy of xenogeneic eASCs for OA and confirmed that IFNγ-primed eASCs may have a therapeutic value for musculoskeletal diseases in veterinary medicine.

## Introduction

Osteoarthritis (OA), also known as degenerative joint disease, is of major concern for human health. It is also one of the most common orthopedic problems seen in horses. Clinical signs of disease are lameness, joint swelling, pain on flexion, or reduced activity, and, with time, these symptoms lead to structural joint alterations. Degradation of articular cartilage is a consequence of its poor capacity to repair and to withstand the cyclic trauma of athletic activity, and this is exacerbated with aging. Current treatments to control inflammation and pain and to stop disease progression include systemic non-steroidal anti-inflammatory drugs (NSAIDs), intra-articular steroids, viscosupplementation, and chondroprotectants (1, 2). A combination of therapies is frequently used to have an additive or synergistic response to injury. However, although no adverse effect has been reported, these approaches would deserve extensive controlled studies to demonstrate efficacy and superiority over unique treatments for precise indications. More recently, the economic impact of musculoskeletal disorders on the horse industry has stimulated many commercial companies to offer stem cell-based regenerative therapies for veterinary purposes (3).

Multipotent mesenchymal stem or stromal cells (MSCs) isolated from bone marrow (BM) or adipose tissue (AT) are the most extensively studied stem cells (4). Techniques for isolating and characterizing equine MSCs (eMSCs) have evolved based on those described for human MSCs (5–8). The main therapeutic applications of MSCs in equine practice are principally for tendinitis and ligament lesions, but OA lesions have become a major challenge. Intra-articular injection of eMSCs or BM concentrates for treatment of OA or cartilage defects has already proved safety with no adverse effect reported (9–11). Interestingly, eMSCs from umbilical cord Wharton’s jelly were shown to be effective in a xenogeneic model of rabbit mild OA (12). However, efficacy of eMSC injection for OA remains controversial with some studies demonstrating molecular [increase of collagen and glycosaminoglycan (GAG) content] and functional improvement (11, 13), while others showing no clinical benefit (14–16).

Mesenchymal stem or stromal cell-based therapy for OA treatment has shown significant results in other animal models [for review, see Ref. (17)]. Moreover, allogeneic or xenogeneic MSCs exert a therapeutic effect similar as syngeneic MSCs, and survival of xenogeneic adipose-derived MSC (ASCs) is not affected by OA environment as compared with healthy environment (18). A majority of studies indeed reports engraftment and function of MSCs across the species barrier with evidence of failure to function in only 6.4% of evaluated cases (19). In the different preclinical models of OA, the anti-inflammatory property of MSCs is thought to have a major role (20–23). Indeed, *in vivo*, it was reported that synovial activation rapidly drives anti-inflammatory and protective effects of intra-articularly injected ASCs, which is reflected by decreased S100A8/A9 alarmins levels (24). We previously showed that MSCs secrete factors that protect OA chondrocytes from hypertrophy, dedifferentiation, inflammation, and apoptosis (25, 26). In addition, MSCs need to be activated to be chondroprotective since their conditioned media do not have any effect on chondrocyte phenotype (27). Demonstration that MSCs require priming for exerting an immunosuppressive function and be more actively recruited has been clearly shown in humans (28, 29). Pre-activation of MSCs occured in presence of interferon-γ (INFγ) and/or tumor necrosis factor (TNF)-α, interleukin (IL1)-α or IL1-β (30), and enhanced their anti-inflammatory effect without influencing their differentiation capacities (31).

While the need for a better evaluation of the functional role of eMSCs using *in vitro* assays is obvious, the possibility to activate the cells for improving their therapeutic effect has not been tested in OA. So, the aims of this study were first to examine the effect of IFNγ-pretreatment on the anti-inflammatory properties of equine ASCs (eASCs) *in vitro* and on the chondroprotective effect of eASCs on cartilage explants and, second, to understand the mechanism of action of eASCs by gene analysis. Finally, the efficacy of IFNγ-pretreated eASCs in OA was evaluated after a single intra-articular injection in the xenogeneic murine model of collagenase-induced OA (CIOA).

## Materials and Methods

### Isolation and Culture of ASC

Equine ASCs were isolated from subcutaneous AT from the horse hip and obtained during surgery by veterinary doctors on tranquilized horses. The protocol was approved by the Ethical Committee of VetAgro Sup (permit number: 69 127 800, certified number: B 69 127 0501). AT was cut into small pieces and digested with 250 U/mL type 1 collagenase (Worthington, Serlabo Technologies, Entraigues) at 37°C for 1.5 h. The stroma vascular fraction was collected by centrifugation (300 *g*, 10 min), and cells were filtered successively through a large sterile filter and, then, through 100 and 70 μm porous membranes (Cell Strainer, BD Biosciences, Le Pont de Claix). Erythrocytes were lysed using red lysis buffer (NH_4_Cl 155 mmol/L, KHCO_3_ 10 mmol/L, EDTA 0.11 mmol/L, pH 7.3). Cell counting and viability were evaluated with calibrated Vicell Beckman Coulter. Cells were plated as passage 0 at the initial density of 4000 cells/cm^2^ in αMEM GlutaMAX™ (ThermoFisher Scientific, Illkirsch) supplemented with 100 U/mL penicillin, 100 mg/mL streptomycin, 10 μg/mL gentamicine/0.25 μg/mL amphotericin B (ThermoFisher scientific), 10% fetal calf serum (FCS, ThermoFisher scientific), and 1 ng/mL basic fibroblast growth factor (bFGF; Sigma-Aldrich, Saint-Quentin-Fallavier). After 1 week, cells were trypsinized and expanded at 2000 cells/cm^2^ till day 14, where ASCs at passage 1 were used. For IFNγ priming, 100 ng/mL of equine IFNγ (R&D Systems, Lille) was added in the culture medium for 24 h before use of the cells. mMSC were isolated from BM of C57BL/6 mice and characterized, as previously described (21).

### Flow Cytometry Analysis

Equine ASCs (200,000 cells) in PBS containing 0.2% bovine serum albumin (BSA) were incubated with different antibodies: non-conjugated ELA-class I (clone CVS22, AbD Serotec, Bio-Rad, Marnes-la-Coquette) or -class II (clone CVS20, Bio-Rad) primary antibodies coupled with Alexa Fluor 488 secondary antibody, PE-conjugated CD29 (clone 4B4LDC9LDH8, Beckman Coulter), CD44 (clone CVS18, Bio-Rad) or CD90 (clone 5E10, Bio-Rad), or the respective isotype control (clone MOPC-21, Bio-Rad) at room temperature for 60 min in the dark. The labeled cells were then analyzed by multiparameter flow cytometry using a LSR II cytometer and the BD FACSDiva™ software V.6.1.3 (BD Biosciences).

### Differentiation of eASCs

Adipogenic differentiation was induced after plating eASCs at the density of 1500 cells/cm^2^ in proliferative medium for 7 days. Medium was changed by adipogenic medium (DMEM-F12, 100 U/mL penicillin, 100 μg/mL streptomycin, 15% Rabbit Serum, 1 μM dexamethasone, 60 μM indomethacin, 10 μg/mL insulin, 0.5 mM IBMX), for 21 days. Adipogenesis was assessed by quantification of adipocyte markers by RT-qPCR, and lipid droplets were visualized by HCS LipidTOX™ Green neutral lipid stain (ThermoFisher Scientific). Osteogenesis was induced by culture at low density (1500 cells/cm^2^) in osteogenic medium (DMEM, 10% FCS, 100 U/mL penicillin, 100 μg/mL streptomycin, 0.1 mM ascorbic acid, 0.1 μM dexamethasone) for 21 days. Osteogenic differentiation was assessed by quantification of osteoblast markers by RT-qPCR and extracellular matrix mineralization detected by alizarin red S staining. Chondrogenic differentiation was induced by pelleting and culturing eASCs (250,000 cells/tube) in chondrogenic medium {high glucose DMEM, 0.1 μM dexamethasone, 1 mM sodium pyruvate, 170 μM ascorbic-2-phosphate acid, 0.35 mM proline, ITS [insulin/transferrin/selenic acid (Lonza, Koln, Germany)], 10 ng/mL human TGF-β3}. Chondrogenesis was assessed by quantification of chondrocyte markers by RT-qPCR and Safranin O–Fast green staining.

### RT-qPCR

Total RNA was extracted from cells using the RNeasy kit (Qiagen, Courtaboeuf). RNA (500 ng) was reverse transcribed using the M-MLV enzyme (ThermoFisher Scientific). qPCR, for evaluating eASC differentiation, was performed using MSC PCR array (Qiagen). Analysis of cytokine expression was done using horse cytokines and chemokines RT^2^ profiler PCR array (Qiagen). Other qPCR analyzes were done with Taqman gene expression assay (Life Technologies, Courtaboeuf). PCR reactions were carried out on 20 ng of cDNA samples according to supplier’s recommendations on a 7500 FAST real-time PCR system (Applied Biosystems) and analyzed with the dedicated software. All values were normalized to GAPDH housekeeping gene and expressed as relative expression or fold change using the respective formulae 2^−ΔCT^ or 2^−ΔΔCt^.

### Equine PBMC Isolation and Proliferative Assay

Equine peripheral blood mononuclear cells (PBMC) were collected from adult allogeneic horses into EDTA-containing tubes *via* jugular venipuncture. Mononuclear cells were isolated from peripheral blood by Ficoll density gradient centrifugation (density 1.077 g/L; Sigma) and suspended in IMDM GlutaMAX™ medium (ThermoFisher Scientific) supplemented with 10% inactivated FCS, 2 mM glutamine, 100 U/mL penicillin, 100 μg/mL streptomycin, 0.1 mM non-essential amino acids, 1 mM sodium pyruvate, 20 mM HEPES (*N*-2-hydroxyethylpiperazine-*N*′-2-ethanesulfonic acid), and 5 × 10^−5^M 2-mercaptoethanol. eASCs were plated in 96-well flat-bottom plates at different densities (10^5^, 10^4^, 10^3^, or 10^2^ cells/well) ± 100 ng/mL equine recombinant INFγ (R&D Systems) at 37°C for 24 h with 5% CO_2_. PBMCs were added at 10^5^ cells/100 μL/well, and proliferation was induced by adding the mitogen phytohemaglutinin (PHA) at 5 μg/mL (Sigma-Aldrich). Unstimulated PBMCs were used as negative control. After 4 days incubation, cultures were pulsed with 5 μCi/mL ^3^H thymidine (Amersham, Buckinghamshire, UK) for 18 h. PBMCs were harvested and thymidine incorporation was expressed as proliferation in counts per minute. The inhibitory effect of eASCs on PBMC proliferation was quantified by subtracting the signal for PHA-stimulated PBMCs from unstimulated PBMCs. Proliferation rate was calculated referring to 100% the value of PHA-stimulated PBMCs.

### Cartilage Explant Culture

Fetlock joints were recovered from the local slaughter house. The joints were aseptically opened with a scalpel in a dissection room to allow access to articular cartilage. Joints were graduated given the OARSI grade (32), and grade 1 joints were selected. A biopsy punch was used to extract 3 mm^3^ explants (average weight of 10 mg) from cartilage. Five explants per well in 24 multi-well plates were cultured in 1 mL of explant culture medium (DMEM, 20 mM Hepes, 5 μg/mL ascorbic acid, ITS, 1% Gentamycin) and incubated at 37°C, in 5% CO_2_ humidified atmosphere. After 2 days, recombinant equine IL-1β (1 ng/mL; R&D Systems, Lille) and recombinant human oncostatin M (10 ng/mL; Invitrogen, Courtaboeuf) were added in the culture medium, and explants were cultured with or without 25,000 or 100,000 eASCs on a transwell membrane (pore 0.4 μm) during 7, 9, or 12 days. eASCs were primed or not with equine IFNγ (100 ng/mL) for 24 h before addition to the explant cultures. Media were changed every 2–3 days and aliquots of supernatants were stored at −20°C until GAG quantification.

### Glycosaminoglycan Quantification

Glycosaminoglycan quantification was performed using Blyscan™ GAG Kit on triplicate wells following supplier’s recommendations (Tebu-Bio, Le Perray en Yvelines).

### Collagenase-Induced Osteoarthritis Mouse Model

Collagenase-induced OA was induced as previously described (18). Animal experimentation was conducted in agreement with the Languedoc-Roussillon Regional Ethics Committee on Animal Experimentation (approval CEEA-LR-10041). All surgery was performed under isoflurane gas anesthesia, and all efforts were made to minimize suffering. Briefly, right knee joints of mice were injected with 1 U type VII collagenase from *Clostridium histolyticum* (Sigma-Aldrich) in 5 μL of saline at day 0 and day 2, causing disruption of the ligaments and local instability of the joint. Groups of 10 mice received at day 7, either saline, mMSC (2 × 10^5^ cells/8 μL), or eASCs [2 × 10^4^ or 2 × 10^5^ cells/8 μL primed or not with IFNγ (100 ng/mL for 24 h)]. Mice were sacrificed at day 42, and the joints were collected, fixed in 4% formalin for 1 week, decalcified in 5% formic acid for 2 weeks, and embedded in paraffin. Knee frontal sections (three sections separated from 140 μm) were cut and stained with Safranin O–Fast Green staining. Histological OA score was determined using the modified Pritzker OARSI score (32). At euthanasia, blood was collected by intracardiac puncture, centrifuged at 1000*g* during 10 min. Sera were recovered and stored at −20°C.

### Serum Cytokine Quantification

Quantification of murine IL1β, IL6, IL10, osteoprotegerin (OPG) and prostaglandin E2 (PGE2) (R&D Systems), CTX2 (ImmunoDiagnostic Systems, Pouilly en Auxois), and cartilage oligomeric matrix protein (COMP) (MD Bioproducts, Zürich) was performed in sera by specific enzyme-linked immunosorbent assays (ELISA) as recommended by the suppliers.

### Statistical Analysis

Data were expressed as the mean ± SEM. Statistical analysis was performed with GraphPad Prism software. The comparison between the different groups was analyzed by one-way analysis of variance (ANOVA) followed by Dunnett *post hoc* test, when data were parametric, or by a Kruskal–Wallis test, when the data distribution was not Gaussian. Comparison between two groups was analyzed with a Mann–Whiney test for non-parametric data. The test used was indicated in the figure legends. A *p* value <0.05 was considered significant.

## Results

### Phenotype and Differentiation Properties of eASCs

**Figure 1:**
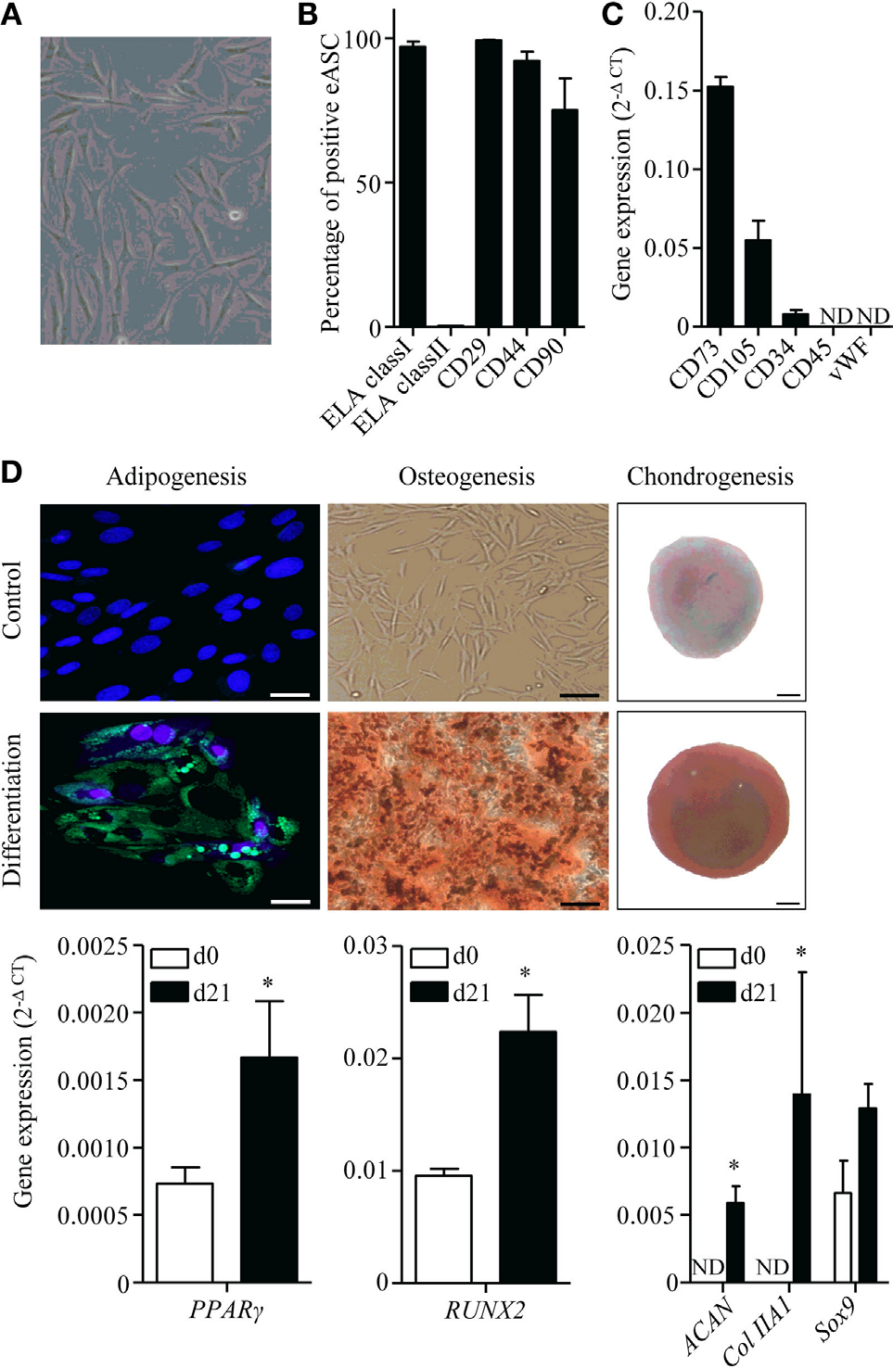
**Characterization of equine ASCs**. **(A)** Representative photomicrograph of eASCs. **(B)** Percentage of eASCs positive for the indicated membrane markers. Results are percentage of positive cells and are expressed as mean ± SEM for three separate experiments. **(C)** Gene expression level of indicated markers in eASCs. Results are relative expression (2^−ΔCT^) and are expressed as mean ± SEM for three separate experiments. **(D)** Differentiation of eASCs: adipogenesis is characterized by the expression of peroxysome proliferator-activated receptor (PPAR)-γ at day 21 (d21) versus d0 and by the visualization of lipid droplets stained with HCS LipidTOX™ Green neutral lipid stain and counterstained with DAPI (lower panel) versus control proliferative medium (upper panel) at d21 (scale bar is 25 μm). Osteogenesis is characterized by the expression of Runx2 at d21 versus d0 and by Alizarin Red S positive staining in differentiation versus proliferative medium at d21 (scale bar is 100 μm). Chondrogenesis is characterized by the expression of Sox9, collagen IIA1 (col IIA1), and aggrecan (Agg) at d21 versus d0 and by Safranin O staining (lower panel) versus undifferentiated control (upper panel) at d21 (scale bar is 100 μm). Results are expressed as relative expression (2^−ΔCT^) and represented as mean ± SEM for three to six independent biological replicates. Data were analyzed using the Mann–Whitney test. *p < 0.05. ND: not detected.

Equine ASCs isolated from subcutaneous AT exhibited a classic fibroblastic morphology at passage 1 (Figure 1A). The majority of cells were positive for CD29, CD44, CD90, and ELA class I markers and negative for ELA class II as detected by flow cytometry (Figure 1B). In absence of available antibodies, expression of ASC markers CD34, CD73, and CD105 was confirmed at the mRNA level, while CD45 hematopoietic and von Willebrand factor (vWF) endothelial markers were not detected (Figure 1C). Under adipogenic conditions, eASCs stored triglycerides in lipid droplets as shown by HCS LipidTOX™ Green neutral lipid staining and the expression of the transcription factor regulating adipogenesis PPARγ, which was significantly increased after 21 days of differentiation compared to day 0 (Figure 1D). eASCs differentiated into osteoblasts, as shown by Alizarin Red S staining and increased Runx2 expression at day 21 (Figure 1D). They also differentiated toward chondrocytes, as suggested by the presence of sulfated GAG evidenced by Safranin O staining and demonstrated by an enhanced expression of Sox9, Aggrecan, and Collagen type II markers (Figure 1D). These results indicated that eASCs possess all the characteristics of mesenchymal stem cells (33).

### Effect of IFNγ Pretreatment on Immunosuppressive and Chondroprotective Properties of eASCs

**Figure 2:**
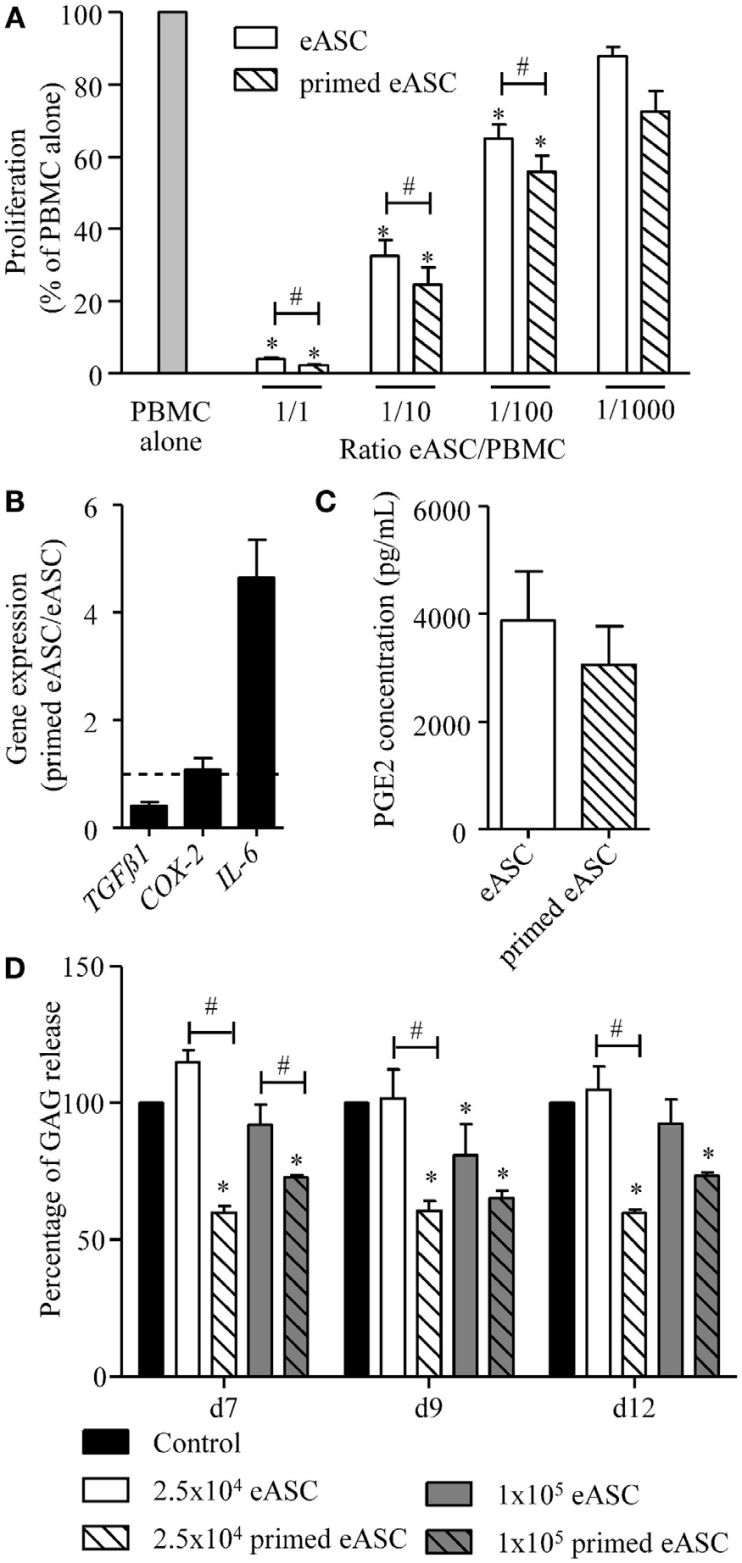
**IFNγ-priming improves the immunosuppressive and chondroprotective properties of eASCs *in vitro***. **(A)** Proliferation of equine PBMCs in presence of naïve eASCs or IFNγ-primed eASCs at different ratios. Results are expressed as the percentage of PHA-induced proliferation of equine PBMCs, which was assigned the value of 100% and represented as mean ± SEM for three independent biological replicates. **(B)** Gene expression level of TGFβ1, COX-2, and IL-6 in eASCs. Gene expression in IFNγ-primed eASCs was normalized to that obtained in naïve eASCs. **(C)** PGE2 concentration in supernatants of naïve and IFNγ-primed eASCs. **(D)** GAG release quantification from cartilage explants incubated with IL1β (1 ng/mL) and Oncostatin M (10 ng/mL) during 7, 9, or 12 days and cocultured with or without 25,000 or 100,000 naïve and IFNγ-primed eASCs (100 ng/mL, 24 h before coculture). Results are expressed as the percentage of GAG release normalized to control condition (explant alone) at each time points and represented as mean ± SEM for 10–14 independent biological replicates. Data were analyzed using the Kruskal–Wallis test followed by Dunn’s test for multiple comparisons for **(A, D)** and using the Mann–Whitney test for **(B,C)** *p < 0.05 in samples versus PBMC alone **(A)** or control **(D)**; ^#^*p* < 0.05 between primed and unprimed samples.

With the rationale in mind that priming eASCs will improve their immunosuppressive properties, we evaluated their capacities to inhibit the proliferation of ePBMCs. eASCs were pretreated or not with IFNγ for 24 h and then cultured with PHA-activated ePBMCs at different ratios for 3 days. When compared with ePBMCs alone, both eASCs- and IFNγ-primed eASCs were able to inhibit T cell proliferation in a dose-dependent manner (Figure 2A). The antiproliferative effect of IFNγ-primed eASCs was, however, significantly higher, whatever the eASC/ePBMC ratio. In order to determine whether this higher suppressive effect of IFNγ-primed eASCs was related to higher paracrine functions, expression of several known immunosuppressive mediators was quantified by RT-qPCR. We noticed that TGFβ1 expression levels were decreased in IFNγ-primed eASCs, while IL6 expression was increased (Figure 2B). No change in expression of COX-2 transcripts or PGE2 secretion was observed (Figures 2B,C).

In parallel, we investigated whether eASCs could exert chondroprotective properties and inhibit cartilage degradation in an *in vitro* model of OA cartilage. We used equine cartilage biopsies cultured with IL1β and Oncostatin M for 12 days to induce cartilage degradation. Cartilage destruction was evaluated by quantifying GAG release in the culture medium. In order to determine the effect of eASCs on cartilage degradation, a coculture assay was designed using a transwell system to avoid eASCs–cartilage contact. Cartilage explants (in the lower part) were cultured with 2.5 × 10^4^ or 10^5^ naïve or IFNγ-primed eASCs (upper part). In these conditions, naïve eASCs did not exert a protective effect on cartilage degradation, even though a significant decrease of GAG release was observed with the dose of 10^5^ eASCs after 9 days of coculture (Figure 2D). By contrast, the two doses of IFNγ-primed eASCs significantly reduced GAG release at the three time points. Interestingly, IFNγ-primed eASCs tended to be more efficient at the lowest dose as compared with the highest dose, but the difference was not significant. Altogether, our data demonstrated that IFNγ-priming of eASCs enhanced their immunosuppressive and chondroprotective function.

### Effect of eASCs in a Xenogeneic Mouse Model of OA

**Figure 3:**
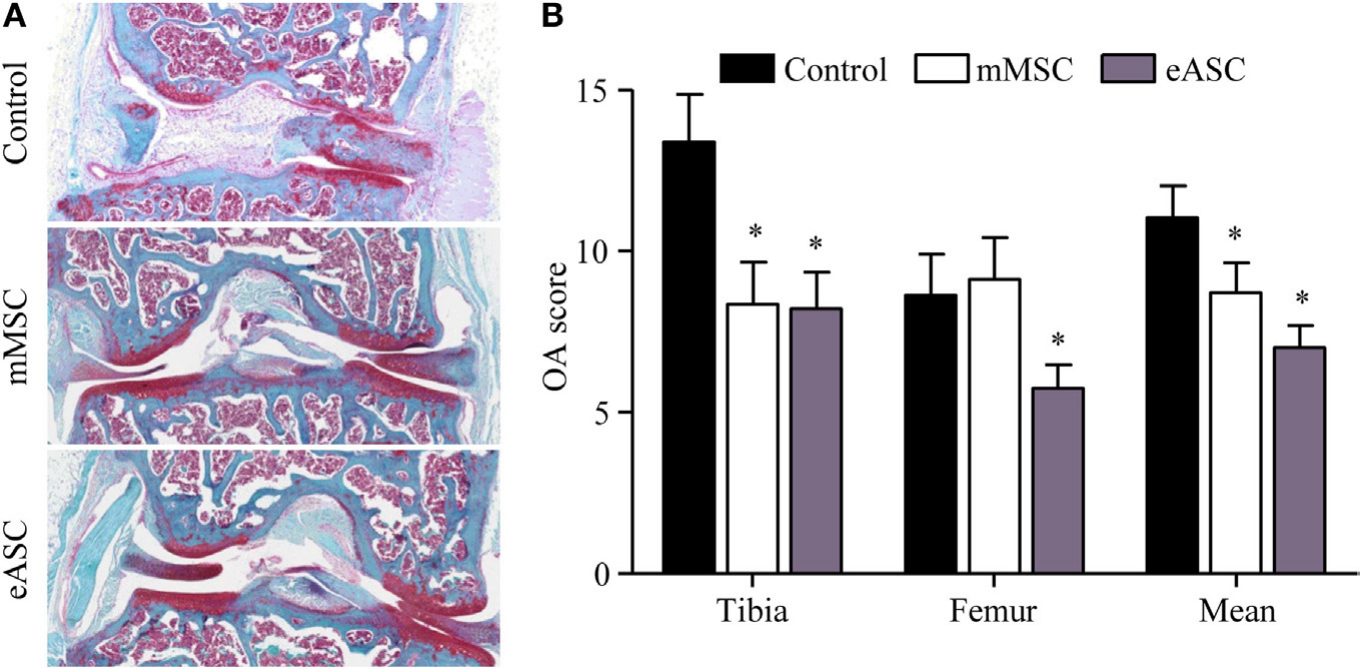
**Xenogeneic eASCs improve OA scores in the collagenase-induced OA mouse model**. OA was induced by intra-articular injection of collagenase in mouse knee joints and the severity of OA was evaluated at day 42. **(A)** Representative photographs of knee joints from control (collagenase alone) (upper), mMSCs-treated (middle), and eASCs-treated (lower) mice. **(B)** Histological OA score of tibia plateaus and femur condyles in the mouse knee joints and mean at euthanasia. Results are expressed as the mean ± SEM, *n* = 10. Data were analyzed using the Kruskal–Wallis test followed by Dunn’s test for multiple comparisons. *p < 0.05.

We, then, investigated whether eASCs could exert a therapeutic effect *in vivo* using a xenogeneic relevant model of OA. We used the CIOA mouse model which is characterized by moderate inflammation of the synovial membrane, osteophyte formation, and cartilage degradation (18). First, we evaluated the effect of intra-articular injection of eASCs (2 × 10^5^ cells) on knee joint OA score by comparison with mMSCs (2 × 10^5^ cells). Histological analysis of knee joint sections revealed protection against cartilage degradation in animals treated either with mMSCs or eASCs as compared with the CIOA control group (Figure 3A). Cartilage protective effect of mMSCs and eASCs was confirmed by scoring both femoral condyles and tibial plateaux in the lateral and medial compartments (Figure 3B). Significantly, lower mean OA scores were obtained after eASCs or mMSCs treatment, and OA score of the eASC group was highly significant compared to control group. These results confirmed that the CIOA mouse model was effective for evaluation of the effect of xenogeneic eASC injection on OA symptoms.

**Figure 4:**
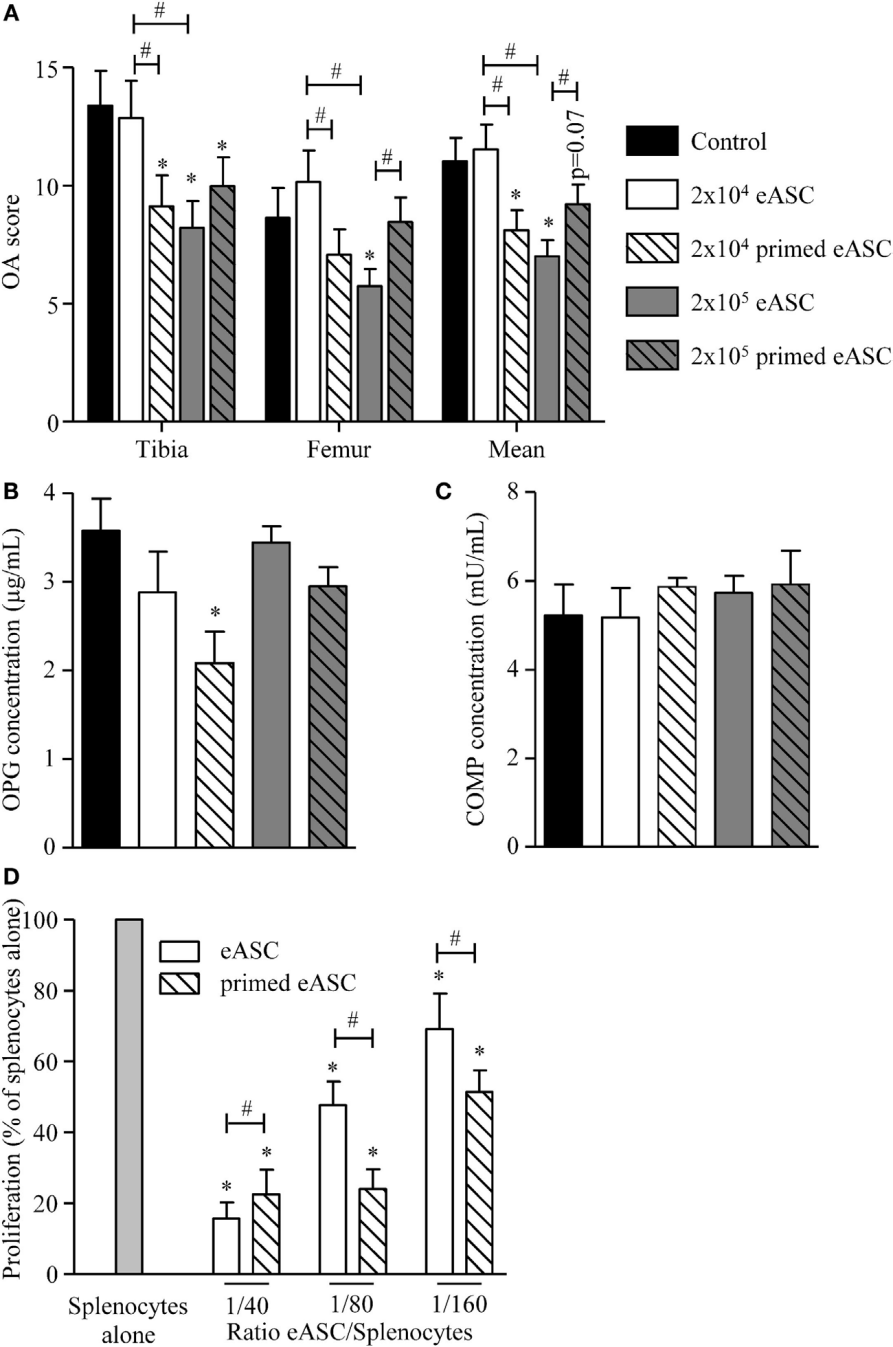
**IFNγ-primed eASCs improve OA score**. **(A)** OA score for tibia plateaus and femur condyles in the knee joint and mean at euthanasia. Results are expressed as the mean ± SEM, *n* = 10. **(B)** Osteoprotegerin (OPG) and **(C)** cartilage oligomeric matrix protein (COMP) concentrations were determined in sera of mice at euthanasia by specific sandwich enzyme-linked immunosorbent assay (ELISA). Results are expressed as the mean ± SEM, *n* = 10. **(D)** Proliferation of murine splenocytes in presence of different ratios of naïve or IFNγ-primed eASCs. Results are expressed as the percentage of ConA-induced proliferation of splenocytes which was assigned the value of 100% and represented as mean ± SEM for three independent biological replicates. Data were analyzed using the Kruskal–Wallis test followed by Dunn’s test for multiple comparisons in A, B, and C and using ANOVA followed by a Dunnett’s test for multiple comparisons in D. *p < 0.05 or *p* = 0.07 compared samples with the control; ^#^*p* < 0.05 compared samples with each other.

We, therefore, compared the effect of naïve and IFNγ-primed eASCs on OA progression. Injection of a high dose of eASCs (2 × 10^5^ cells) significantly improved the OA score, while the low dose (2 × 10^4^ eASCs) had no effect (Figure 4A). By contrast, both doses of IFNγ-primed eASCs reduced the OA score, but only the lowest dose of IFNγ-primed eASCs induced a significant decrease. Of importance, 2 × 10^4^ IFNγ-primed eASCs were as efficient as 2 × 10^5^ naïve eASCs to protect cartilage from degradation. The therapeutic effect of eASCs was confirmed by measuring the level of OPG, a bone metabolism marker, in the sera of mice at euthanasia. It was significantly decreased in mice treated with 2 × 10^4^ IFNγ-primed eASCs (Figure 4B). Levels of COMP, a marker of cartilage turnover, did not change in the sera of treated mice compared with control group (Figure 4C), and IL6, IL10, IL1β, and C-terminal cross-linked telopeptide of type II collagen (CTX2) were not detected in the sera of mice (data not shown).

Finally, we evaluated whether naïve or IFNγ-primed eASCs could inhibit murine T cell proliferation since the CIOA mouse model is known to be associated with local moderate inflammation. We, therefore, tested naïve and IFNγ-primed eASCs on murine splenocytes in a proliferative assay. Both eASCs significantly decreased splenocyte proliferation in a dose-dependent manner, and this inhibitory effect was higher with IFNγ-primed eASCs at the lowest ratios of eASCs/splenocytes (Figure 4D). These data suggested that the effect of eASCs *in vivo* could be mediated at least in part by their inhibitory role on inflammatory cells. Overall, we present evidence that eASCs were efficient to reduce the clinical score of OA in the xenogeneic CIOA model, and IFNγ-pretreatment increased their immunosuppressive and chondroprotective functions.

### Effects of IFNγ Priming on the Cytokine Profile of eASCs

**Figure 5:**
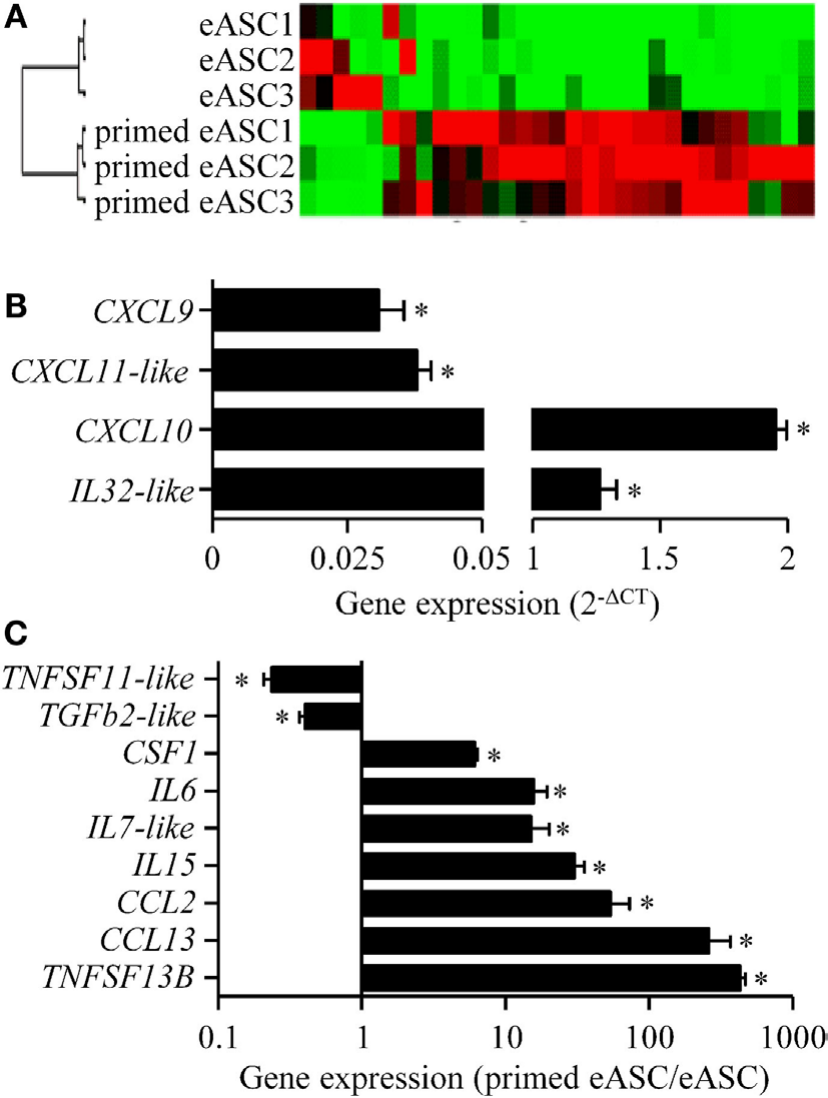
**Effect of IFNγ-priming on the inflammatory gene profile of eASCs**. Gene array analysis of inflammatory cytokines and chemokines mRNA compared naïve and IFNγ-primed eASCs. **(A)** Hierarchical clustering comparing naïve or IFNγ-primed eASCs, **(B)** induced gene expression levels in IFNγ-primed eASCs expressed as relative expression (2^−ΔCT^), **(C)** significantly modulated gene expression levels in IFNγ-primed eASCs. Results are represented as mean ± SEM for three independent biological replicates. Data were analyzed using the Mann–Whitney test. *p < 0.05.

In order to understand the effect of IFNγ priming on the immunosuppressive properties of eASCs, a gene expression analysis was performed on 89 genes related to cytokine or chemokine families. Hierarchical clustering analysis revealed that the gene expression pattern of IFNγ-primed eASCs greatly differed from that of naïve eASCs (Figure 5A). IFNγ priming modified the transcriptomic program of eASCs by significantly dysregulating 13 genes. Gene expression of CXCL9, CXCL11-like, IL32-like, and CXCL10 was significantly induced in IFNγ-primed eASCs compared with naïve eASCs (Figure 5B). In parallel, gene expression of CSF1, IL6, IL7-like, IL-15, CCL2, CCL13, and TNFS13B was significantly upregulated in IFNγ-primed eASCs, while expression of TNFSF11-like and TGFβ2-like was downregulated as compared with naïve eASCs (Figure 5C). Indeed, the cytokine/chemokine profile of eASCs was dramatically altered by IFNγ priming.

## Discussion

The present study demonstrated the feasibility of using a mouse OA model for assessing the efficacy of eASC-based therapy and developing eASC pretreatment strategies to enhance their therapeutic potential.

Equine athletes are subject to a number of musculoskeletal injuries, some of which will lead to degenerative joint diseases. The lack of effective treatment to stop disease progression causes horses to retire early from competition. The current challenge is, therefore, to find a treatment allowing sport horses to keep or increase their athletic level. However, evaluating the effectiveness of novel therapies in an equine model of OA is time and cost consuming. The possibility to rely on a small-animal model that is relevant for reproducing the main characteristics of the disease should allow the optimization of the optimal doses, timings, and pretreatment options in a rapid prescreening step for further upgrading to larger animals.

Because the main effect of ASCs in OA seems to be related to their anti-inflammatory functions (17), the choice of the mouse model to be used for evaluating eASC efficacy is of importance. Indeed, local administration of murine ASCs after destabilization of medial meniscus (DMM) model had no effect on OA pathology (24). By contrast, murine ASC treatment was shown to be efficient in the CIOA model, which is characterized by a highly activated synovial lining containing inflammatory macrophages, and efficacy of ASCs was related to the degree of synovial activation (23, 24). In experimental models, synovitis was, therefore, shown to be essential to mediate the anti-inflammatory function of ASCs. We confirmed in the present study the usefulness of the CIOA model to assess efficacy of ASC-based therapy in a xenogeneic approach as previously demonstrated with human ASCs (18). The feasibility of using a xenogeneic model to investigate the role of eASCs in OA was also recently validated in another study. IA injection of eMSCs isolated from umbilical cord Wharton’s jelly partly prevented OA signs in the rabbit model of medial meniscal release (MMR). MSCs were shown to target the synovium by modulating the expression of matrix-degrading enzymes in favor of an anti-catabolic environment (12). The interest of using the mouse model of OA, however, is the cost effectiveness and the availability of various reagents or genetic models that allow easier evaluation of mechanisms of action. Furthermore, our results demonstrated that mice receiving eASCs did not develop any adverse effects confirming the safety of IA injection of xenogeneic ASCs for the treatment of OA (18). In addition, the CIOA mouse model could be useful to determine the potency of different batches of eASCs *in vivo* in terms of anti-inflammatory and chondroprotective functions. This could be of interest for veterinary stem cell companies, which already provide allogeneic eASC or eMSC banking, in order to evaluate donor cell variability.

Besides providing evidence that CIOA model is a suitable model to assess eASC therapy, the second objective of this study was to demonstrate the efficacy of IFNγ pretreatment for improving the therapeutic potential of eASCs. Priming of MSCs by inflammatory signals is required for their homing and anti-inflammatory functions and could, therefore, enhance therapeutic efficacy [for review, see Ref. (29, 34)]. Here, we demonstrated that IFNγ-pretreatment for 24 h improved the *in vitro* capacities of eASCs to inhibit the proliferation of T lymphocytes and to reduce the catabolic activity of chondrocytes. This effect could be explained by the modulation of the secretome, as evidenced at the gene expression level. Indeed, IFNγ-pretreatment induced or upregulated the expression of several chemokines, most notably CXCL9, CXCL10, and CXCL11, which are ligands for CXCR3, the T cell-specific chemokine receptor. The critical role of these chemokines in the immunosuppressive properties of MSCs was shown using neutralizing antibodies against CXCR3 that prevented the ability of MSCs to recruit leukocytes and inhibit their proliferation (30). Among upregulated cytokines, IL15 and IL6 also participate to the anti-inflammatory functions of MSCs. IL15 produced by MSCs has been shown to attract NK cells, alter their phenotype, suppress their proliferation and cytokine secretion ability, and decreased their cytotoxicity (35). IL6 has also been demonstrated to act on PGE2 secretion, neutrophil apoptosis, and macrophage and dendritic cells polarization toward a tolerogenic phenotype (21, 36). Gene expression analysis also evidenced TGFβ2 downregulation in IFNγ-primed eASCs. TGFβ2 is a known chondrogenic factor, but its expression is also related with the loss of immunosuppressive effect (37, 38). The role of other up or downregulated mediators (IL32-like, CSF1, IL7-like, CCL2, CCL13, TNFS13B, TNFSF11-like) in the immunosuppressive potential of MSCs is less known but, in view of the high levels of upregulation after IFNγ-priming, these mediators warrant further investigation. All together, these data strongly suggest that IFNγ-priming of eASCs enhanced their immunosuppressive abilities and could reduce synovial inflammation as observed *in vivo*.

Moreover, our results demonstrated that IFNγ-primed eASCs display protective effect on cartilage degradation *in vitro*, while naïve eASCs did not. These results are in concordance with previous data reporting that inflammatory signals enhanced the beneficial role of MSCs on OA chondrocytes. In a 2D coculture model, hASCs were shown to decrease the secretion of several inflammatory factors when chondrocytes from OA patients expressed high levels of inflammatory molecules, but not when chondrocytes were poorly inflammatory (25). In addition, conditioned media from unprimed hASCs did not impact chondrocyte phenotype demonstrating the importance of inflammatory priming for chondroprotection (27). The inhibition of catabolic activity observed in our experiments by the decrease of GAG release when eASCs were cocultured with cartilage explants could be explained by reduction of metalloproteinases and ADAMTS expression in chondrocytes, as recently reported (39). Together with reduced catabolic activity in an inflammatory environment, the authors also showed ASC-induced autophagy that is a protective mechanism in normal cartilage, which is downregulated with OA (40). We also provided some evidence that eASCs participated to subchondral bone remodeling *in vivo* as suggested by OPG downregulation in the sera of mice with CIOA after treatment. Decreased levels of OPG could be related with increased osteoclastogenesis and resorption of bone sclerosis, which is one characteristic of OA. Indeed, in humans, presence of subchondral bone osteoblasts with low and high OPG levels was proposed to reflect different stages of attempts to repair the damaged tissue in this disease: an increase in bone resorption followed by bone formation (41).

Finally, the finding that IFNγ-pretreatment improved eASCs efficiency *in vivo* confirmed the importance of ASC priming by inflammatory signals to exert a full activity. Of importance, we, therein, demonstrated a similar efficacy for 2 × 10^4^ IFNγ-primed eASCs and 2 × 10^5^ naïve eASCs highlighting the possibility to enhance the therapeutic potential of eASCs with low anti-inflammatory activity. This is relevant for autologous MSC-based therapies, where cells from diseased or old animals could be altered in their suppressive function (42). It may also be of interest to reduce the number of MSCs required per subject and, therefore, the cost of good manufacturing practice (GMP)-grade production. The data further suggested the importance of ASC pretreatment to overcome absence or low level of *in vivo* priming. Indeed, the immunoregulatory function of MSCs is highly plastic. MSCs can be rendered immunosuppressive in the presence of strong inflammation, while weak inflammation causes MSCs to enhance the immune response (43). Different responses of MSCs to different levels of local inflammation will, therefore, influence MSC activity, which may explain the different outcomes observed in various pathologies or following diverse timing of injection for the same disease (21). Indeed, pretreatment of MSCs with IFNγ has already been shown to significantly increase therapeutic effects in mice suffering from graft-versus-host disease or hepatitis (44, 45). Such pretreatment approaches may mimic the pathological inflammatory environments and avoid risks of unresponsiveness. This has still to be validated in clinical trials as a way to improve MSC-based clinical therapies.

In conclusion, we reported that IFNγ-pretreatment of eASCs greatly improved their therapeutic efficacy in terms of immunosuppressive and chondroprotective functions for OA treatment. We also demonstrated the relevance of using the CIOA mouse model to evaluate the role of naïve or pretreated xenogeneic eASCs in a cost-effective way before extrapolation to an equine-specific OA model we have recently developed (2).

## Author Contributions

All authors contributed in drafting the article and all authors approved the final version. Study conception and design: GP, CJ, DN, and RS. Acquisition of data: IB, MJ, MM, DO, GR, ST, and KT. Analysis and interpretation of data: IB, MJ, OL, MM, DN, DO, GP, GR, RS, ST, and KT. Drafting the article or revising it critically for important intellectual content: IB, CJ, MJ, OL, MM, DN, DO, GP, GR, RS, ST, and KT. Final approval of the version of the article to be published: IB, CJ, MJ, OL, MM, DN, DO, GP, GR, RS, ST, and KT.

## Conflict of Interest Statement

GR, GP, IB, ST, DO, MJ, and RS are employees of SANOFI-AVENTIS. The remaining authors declare that the research was conducted in the absence of any commercial or financial relationships that could be construed as a potential conflict of interest.
